# Immunomodulation with dendritic cells and donor lymphocyte infusion converge to induce graft *vs* neuroblastoma reactions without GVHD after allogeneic bone marrow transplantation

**DOI:** 10.1038/sj.bjc.6605924

**Published:** 2010-10-26

**Authors:** S Ash, J Stein, N Askenasy, I Yaniv

**Affiliations:** 1 Department of Pediatric Hematology-Oncology, Schneider Children's Medical Center of Israel, Petach Tikva 49202, Israel; 2 Zaizov Cancer Immunotherapy Laboratory, Schneider Children's Medical Center of Israel, Petach Tikva 49202, Israel; 3 Pediatric Bone Marrow Transplant Unit, Schneider Children's Medical Center of Israel, Petach Tikva 49202, Israel; 4 Frankel Laboratory of Experimental Bone Marrow Transplantation, Schneider Children's Medical Center of Israel, 14 Kaplan Street, Petach Tikva 49202, Israel

**Keywords:** neuroblastoma, allogeneic bone marrow transplantation, dendritic cells, donor lymphocyte infusion, graft *vs* tumour reaction, graft *vs* host disease

## Abstract

**Background:**

Mounting evidence points to the efficacy of donor lymphocyte infusion (DLI) and immunisation with tumour-pulsed dendritic cells (DC) in generating graft *vs* leukaemia reactions after allogeneic bone marrow transplantation (BMT). We assessed the efficacy of DLI and DC in generating potent graft *vs* neuroblastoma tumour (GVT) reactions following allogeneic BMT.

**Methods:**

Mice bearing congenic (H2K^a^) Neuro-2a tumours were grafted with allogeneic (H2K^b^) T-cell-depleted bone marrow cells. Tumour-pulsed donor DC (DC^Neuro2a^) were inoculated (on day +7) in conjunction with donor (H2K^b^) and haploidentical (H2K^a/b^) lymphocytes.

**Results:**

Murine Neuro-2a cells elicit immune reactions as efficient as B lymphoma in major histocompatibility complex antigen-disparate mice. Lymphopenia induced by conditioning facilitates GVT, and transition to adaptive immunity is enhanced by simultaneous infusion of and DC^Neuro2a^ and lymphocytes devoid of graft *vs* host (GVH) activity (H2K^a/b^). In variance, the efficacy of DC-mediated immunomodulation was diminished by severe graft *vs* host disease (GVHD), showing mechanistic dissociation and antagonising potential to GVT.

**Conclsions:**

The GVHD is not a prerequisite to induce GVT reactivity after allogeneic BMT, but is rather detrimental to induction of anti-tumour immunity by DC-mediated immunomodulation. Simultaneous inoculation of tumour-pulsed donor DC and DLI synergise in stimulation of potent GVT reactions to the extent of eradication of established NB tumours.

Neuroblastoma (NB) is a low immunogenic solid tumour that expresses low levels of major histocompatibility complex (MHC) antigens and few known specific antigens, most of which are shared with other tissues of ectodermal origin (Cooper *et al*, 1990; Coze *et al*, 1995; Prigione *et al*, 2004; Wolfl *et al*, 2005). Considering the poor outcome of surgery and chemoradiotherapy in paediatric patients with high-risk NB, immune-based therapeutic approaches are being pursued (Verneris and Wagner, 2007; Navid *et al*, 2009). For example, dendritic cells (DCs) are crucial ingredients towards induction and propagation of tumour antigen-specific immune reactions (Banchereau and Palucka, 2005), endowed with the capacity to sensitise the immune system against NB tumours *ex vivo* (Valteau-Couanet *et al*, 2002) and *in vivo* (Geiger *et al*, 2000; Caruso *et al*, 2005). Bone marrow transplantation (BMT) evolves as a standard therapeutic procedure aiming to maximise the efficacy and intensity of anti-tumour immunity (Fish and Grupp, 2008; Ladenstein *et al*, 2008). Consequently, immunomodulatory approaches have adopted to the transplant setting, consisting either of vaccination with tumour-pulsed DC (Asavaroengchai *et al*, 2002) or donor lymphocyte infusion (DLI) under conditions of controlled graft *vs* host disease (GVHD, Naparstek *et al*, 1996; Slavin, 2001).

Autologous BMT for immunohaematopietic reconstitution after aggressive chemoradiotherapy have yielded modest results in the absence of additional immunomodulation (Yaniv *et al*, 1990; Kanold *et al*, 2000). The modest efficacy might be attributed to the absence of graft *vs* tumour (GVT) reactions (Slavin *et al*, 2000), which is more efficiently attained by allogeneic and haploidentical BMT (Demirer *et al*, 2008; Kanold *et al*, 2008). Although allogeneic BMT *per se* is endowed with the capacity to generate anti-tumour immune reactivity (Ash *et al*, 2009), significant reduction and eradication of even small-sized NB tumours requires substantial post-transplant immunomodulation (Anderson *et al*, 2000; Borrello *et al*, 2000; Teshima *et al*, 2001; Asavaroengchai *et al*, 2002). Accordingly, DCs have been efficiently implemented to foster immunity against haematological malignancies after allogeneic transplantation (Li and Waller, 2004; Reddy *et al*, 2005; Tatsuta *et al*, 2009). However, haematological malignancies can elicit anti-tumour immunity through selective expression of minor histocompatibility antigens (Riddell *et al*, 2002), which are largely absent in solid tumours (Ringdén *et al*, 2009). In addition to effective immunisation against solid tumours using tumour-pulsed DC (Geiger *et al*, 2000; Caruso *et al*, 2005), recent evidence points to the superior activity of DC after allogeneic BMT (Moyer *et al*, 2006). Successful *ex vivo* sensitisation and expansion of tumour-reactive lymphocytes attribute DC a stimulatory effect (Xia *et al*, 2006; Ghosh *et al*, 2009), which can be achieved also by DLI (Eto *et al*, 2008; Kamiryo *et al*, 2009; Yoshida *et al*, 2009). Accordingly, the interaction between DC and lymphocytes (Chakraverty *et al*, 2006) has been implemented in a number of ways, including immunomodulation of the donors (Asavaroengchai *et al*, 2004) and immunomodulation of the recipients (Li and Waller, 2004; Moyer *et al*, 2006), although the relative roles of host and donor DC in GVT and GVH reactions is under debate (Shlomchik *et al*, 1999; Matte *et al*, 2004; Xia *et al*, 2006; Chakraverty and Sykes, 2007).

These data indicate that cytotoxic lymphocytes are effectively propagated by tumour-pulsed DC, with effective anti-leukaemia effects attained by post-transplant administration of lymphocytes and DC. Following the demonstration that tumour-pulsed donor DC effectively suppress NB growth after allogeneic BMT (S Ash, I Yaniv, N Askenasy, J Stein, unpublished data), it is questioned whether their effect can be amplified by DLI to elicit potent GVT reactions against this solid tumour. In variance from previous studies aiming to decipher the mechanism of interaction between antigen-presenting cells and lymphocytes (Shlomchik *et al*, 1999; Li and Waller, 2004; Matte *et al*, 2004; Chakraverty *et al*, 2006; Xia *et al*, 2006), we adopted a clinically relevant experimental model of post-transplant immunomodulation of tumour bearing mice using donor cells. We found that murine NB is an immunogenic tumour submitted to immune surveillance after allogeneic BMT through GVT reactivity that is independent of GVHD. Post-transplant immunisation with tumour-pulsed antigen-presenting cell in conjunction with lymphocytes devoid of GVH activity fosters significantly GVT reactivity to the extent of eradication of established tumours.

## Materials and methods

### Animal model

Mice used in this study were A/J (H2K^a^, CD45.2), C57BL/6 (H2K^b^, CD45.2), B6.SJL-Ptprc^a^ Pepc^b^/BoyJ (H2K^b^, CD45.1), inbred A:C57BL/6 F1 chimeras (H2K^a/b^, CD45.2) and non-obese diabetic/severe combined immunodeficiency (NOD/SCID) mice purchased from Jackson Laboratories (Bar Harbor, ME, USA). Mice were housed in a barrier facility in accordance with the guidelines of the Institutional Animal Care and Use Committee.

### Tumour cells

Neuro-2a cells murine NB (H2K^a^) and A20 B-cell lymphoma (H2K^d^) were obtained from the American Type Culture Collection (ATCC, Manassas, VA, USA). Cells were cultured to maximum 12 passages as previously described (Ash *et al*, 2009). Subcutaneous tumours were induced by implantation of 10^6^ Neuro-2a cells in 100 *μ*l of phosphate-buffered saline (PBS). Tumour growth was measured with a caliper and the volume (mm^3^) was calculated according to: (width^2^ × length × 0.4).

### Bone marrow cell preparation

Whole bone marrow cells (wBMCs) were harvested from the femurs and tibia of donors, and low-density cells were collected as previously described (Askenasy and Farkas, 2003). Immunomagnetic T-cell depletion was performed by incubating for 45 min at 4°C with saturating amounts of biotinylated anti-mouse monoclonal antibodies (mAbs) specific to CD4, CD5 and CD8 (hybridoma cell cultures, ATCC). The mAb-coated cells were washed twice with PBS (Beit Haemek, Israel) containing 2% FCS (Biological Industries, Beit Haemek, Israel) and were incubated with sheep-anti-rat IgG conjugated to M-450 magnetic beads at a ratio of four beads per cell (Dynal Inc., Lake Success, NY, USA). Conjugated cells were precipitated by exposure to a magnetic field. The efficiency of T-cell depletion was reassessed by flow cytometry using a cocktail of primary labelled mAb against the T-cell markers listed above.

### Splenocyte preparation

Spleens were harvested from mice, minced, passed through 40-*μ*m mesh and dispersed into single-cell suspensions in PBS (Kaminitz *et al*, 2009). Red blood cells were lysed with medium containing 0.83% ammonium chloride, 0.1% potassium bicarbonate and 0.03% disodium EDTA. After 4 min, the reaction was arrested with excess of ice-cold PBS. T cells were enriched by elution through a cotton wool column (preferential retention of B lymphocytes and myeloid cells by differential charge than the eluted T cells), or immunomagnetic depletion using hybridoma-derived antibodies against GR-1, Mac-1 and B220 (ATCC). This procedure routinely provides an enriched population of 70–80% T cells.

### Dendritic cells

Mononuclear cells were harvested from bone marrow samples by gradient centrifugation over murine Lympholyte (Cedarlane, Burlington, Ontario, Canada) and cultured (10^6^ cells per ml) in low-LPS RPMI 1640 (<10 pg ml^–1^) supplemented with 10% FBS, 1% L-glutamine, 1% sodium pyruvate, 1% *α*-MEM non-essential amino acids, 0.1% Hepes buffer and 1% Pen/Strep (Biological Industries; Gibco BRL, Grand Island, NY, USA; Sigma, St Louis, MO, USA). The medium was supplemented with 50 ng ml^–1^ mouse recombinant (mr) granulocyte macrophage colony-stimulating factor (mrGM-CSF) and 10 ng ml^–1^ interleukin-4 (mrIL-4) on intermittent days (PeproTech, Rocky Hill, NJ, USA). Naïve DCs were incubated for additional 24 h in DC medium without growth factors. Pulsing with tumour antigens was performed by co-incubation of DC with tumour lysate at an approximate DC/tumour cell ratio of 3 : 1 (Ash *et al*, 2009). Tumour lysate is collected from the soluble fraction of Neuro-2a cells detached by trypsin, after three cycles of freezing in liquid nitrogen (2 min) and thawing at 37°C (4 min) in PBS.

### Conditioning and transplantation

Recipients were conditioned with total body irradiation at 700 rad using an X-ray irradiator (Rad Source 2000; Rad Source Technologies Inc., Alpharetta, GA, USA) at a rate of 106 rad min^–1^. After 6 h, cells were injected into the lateral tail vein in 200 *μ*l PBS. Naïve and tumour-pulsed DCs were inoculated subcutaneously adjacent to the tumour on day +7 after transplantation, and splenocytes were adoptively transferred intravenously.

### Flow cytometry

Blood was collected in heparinised serum vials in 200 *μ*l M199, centrifuged over 1.5 ml lymphocyte separation media (Cedarlane), and red blood cells were lysed. Nucleated cells were incubated for 45 min at 4°C with phycoerythrin (PE)-anti-H2K^b^ (Caltag, Carlsbad, CA, USA) and fluorescein isothiocyanate (FITC)-anti-H2K^k^ mAb cross-reactive with H2K^a^ (eBioscience, San Diego, CA, USA). Minor antigen disparity was assed using CD45.1-PE and CD45.2-FITC antibodies (eBioscience). T cells were quantified using CD4-allophycocyanin and CD8-FITC antibodies (BD Pharmingen, San Diego, CA, USA). Measurements were performed with a Vantage SE flow cytometer (Becton Dickinson, Franklin Lakes, NJ, USA). Positive staining was determined on a log scale, normalised with control cells stained with isotype control mAb.

### Histology

Skin and liver were collected from mice killed by CO_2_ asphyxiation, and were fixed in ice-cold PBS containing 1.5% fresh paraformaldehyde for 2 h at 0–4°C. Tissues were embedded in OCT (Sakura Finetek, Torrance, CA, USA), frozen in isopentane suspended in liquid nitrogen, sectioned (3–6 *μ*m) with a Cryotome (Termo Shandon, Cheshire, UK) and stained with haematoxylin and eosin.

### GVH disease

Graft *vs* host disease was assessed using a semiquantitative clinical scale including weight loss, posture (hyperkeratosis of the foot pads impairs movement), activity, diffuse erythema (particularly of the ear) or dermatitis, and diarrhoea. GVHD was validated by histology in haematoxylin and eosin sections of the ear and liver according to: 0 – no infiltration, 1 – scarce infiltrates, 2 – patchy infiltration and 3 – diffuse infiltration with deterioration of tissue structure. The modified score for the liver (Fukui *et al*, 2007) is based on initial histological definition of GVHD pathology (Blazar *et al*, 1993; Cooke *et al*, 1998).

### Proliferation assays

Splenocytes were layered in plastic dishes for 45 min and non-adherent cells were labelled with 2.5 *μ*M 5-(and-6-)-carboxyfluorescein diacetate succinimidyl ester (CFSE; Molecular Probes, Eugene, OR, USA). Triplicate cultures were harvested after 3–5 days, evaluated for CFSE dilution by flow cytometry and data were quantified using the ModFit software (Verity Software House, Topsham, ME, USA). In some cases the cells were exposed to mitogenic stimulation with 5 *μ*M concanavalin A (Biological Industries).

### Cytotoxic assays

Effector splenocytes harvested from naïve mice and chimeras were lysed and passed through wool mesh to enrich for T lymphocytes (∼70%). These cells were incubated with 5 × 10^5^ Neuro-2a target cells for 7 h at 37°C in 150 *μ*l at 1 : 10–1 : 100 target/effector ratios. Cytolysis was quantified by lactate dehydrogenase (eBioscience) release and normalised for background values.

### Statistical analysis

Data are presented as means±s.d. for each experimental protocol. Results in each experimental group were evaluated for reproducibility by linear regression of duplicate measurements. Differences between the experimental protocols were estimated with a *post hoc* Scheffe *t*-test and significance was considered at *P*<0.05.

## Results

### Immunogenicity of Neuro-2a cells

**Figure 1:**
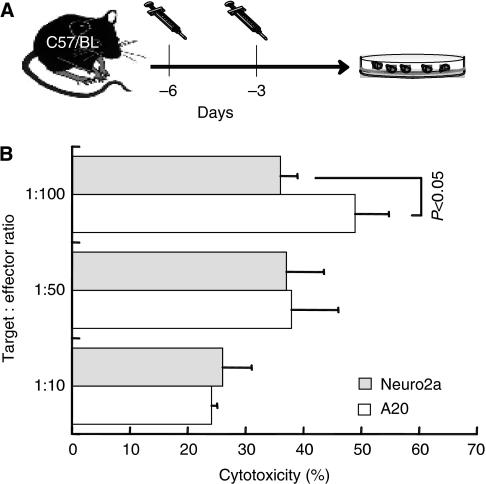
Neuro-2a cells elicit immune reactions. (**A**) C57/BL mice (H2K^b^) were immunised with two intravenous injections of 3 × 10^6^ Neuro-2a (H2K^a^) or A20 cells (H2K^d^) at 3-day intervals and cytolytic responses were evaluated after 3 days. (**B**) Target Neuro-2a (*n*=6) and A20 cells (*n*=5) were exposed to lymphocytes from respectively immunised mice at various target/effector ratios to determine lysis according to lactate dehydrogenase (LDH) release. Data represent means±s.d.

The Neuro-2a cell line is considered to be particularly low immunogenic, as a general feature of human NB. To determine the relative efficacy of tumour antigen presentation by Neuro-2a cells, cytolytic responses were compared with the murine A20 lymphoma cell line. B6 mice (H2K^b^) were immunised twice at 3-day interval, and lymphocytes were co-incubated with target cells (Figure 1A). Neuro-2a cells (H2K^a^) were gradually killed at decreasing target/effector ratios and were comparable to lysis of A20 (H2K^d^, Figure 1B), with significant difference only at the lowest target/effector ratio (*P*<0.05). Therefore, the Neuro-2a cell line elicits effective immune responses comparable to an immunogenic cell line (Asavaroengchai *et al*, 2002, 2004).

### Lymphopenia is favourable to the induction of GVT reactivity

**Figure 2:**
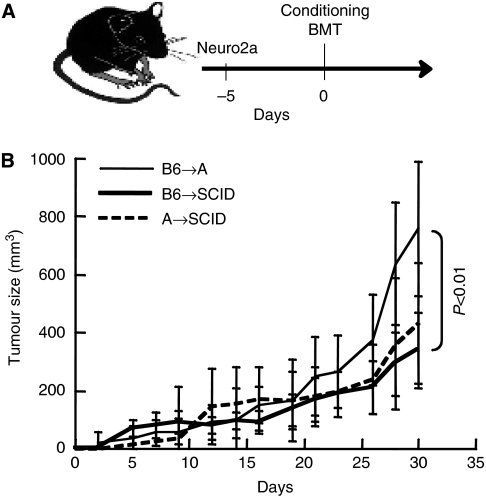
Tumour growth in congenic and immunocompromised mice. (**A**) Mice were implanted with subcutaneous Neuro-2a cells and after 5 days were conditioned and grafted with 5 × 10^6^ whole bone marrow cells (BMCs). Immunocompetent mice were irradiated (700 rad) and immunocompromised non-obese diabetic/severe combined immunodeficiency (NOD/SCID) mice were conditioned with busulfan (2 × 25 *μ*g g^–1^). (**B**) Tumour growth in allografted mice congenic to the tumour (H2K^a^, *n*=19) and NOD/SCID mice (H2K^g7^) grafted with whole BMCs from B6 (H2K^b^, *n*=9) and A (H2K^a^, *n*=7) donors.

Tumour growth in congenic mice is limited as compared with immunocompromised mice, suggesting that Neuro-2a is submitted to immune surveillance in immunocompetent mice (Ash *et al*, 2009). Furthermore, transition to adaptive immunity by either syngeneic or allogeneic BMT suppressed tumour growth, suggesting that a state of immune activation is beneficial to GVH reactivity. To evaluate whether lymphopenia actually promotes GVT reactivity (Borrello *et al*, 2000; Hu *et al*, 2002), tumour growth in reconstituted NOD/SCID mice was compared with allogeneic transplants (Figure 2A): mice were implanted with subcutaneous tumours and after 5 days were conditioned and grafted with 5 × 10^6^ allogeneic BMC. NOD/SCID mice were conditioned with busulfan, which is significantly less immunosuppressive as compared with total body irradiation (Askenasy and Farkas, 2003). Tumour growth was significantly suppressed by transplantation of allogeneic whole BMC from H2K^a^ and K2K^b^ donors into NOD/SCID mice (*P*<0.01, Figure 2B), consistent with avid repopulation of the lymphopenic organs. Notably, residual host immune cells are detected in lymphoid organs of allografted immunocompetent mice after myeloablative irradiation (Kaminitz *et al*, 2009), whereas in NOD/SCID mice the lymphoid organs were full donor (not shown). Therefore, both intrinsic lymphopenia in NOD/SCID mice and radiation-induced lymphodepletion facilitate GVT reactivity.

### Relationship between GVT and GVHD

**Figure 3:**
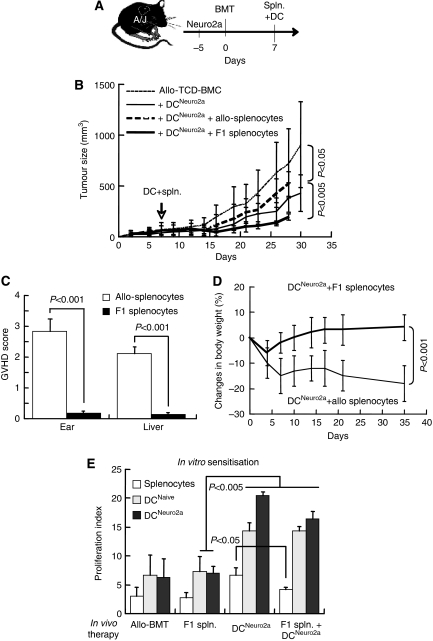
Adoptive transfer of dendritic cells (DCs) and lymphocytes in allografted mice bearing tumours. (**A**) Experimental design of 5 × 10^6^ allogeneic (H2K^b^ → H2K^a^) T-cell-depleted bone marrow cell (TCD-BMC) transplantation into irradiated (700 rad) mice implanted with subcutaneous tumours 5 days earlier. On day +7 the mice were inoculated subcutaneously with tumour-pulsed DC (DC^Neuro2a^) in conjunction with intravenous infusion of 2 × 10^7^ donor (H2K^b^) or F1 (H2K^a/b^) splenocytes (spln.). (**B**) Tumour growth in allografted mice (*n*=19), recipients of DC^Neuro2a^ alone (*n*=17) and in conjunction with allogeneic (*n*=6) or F1 splenocytes (*n*=11). (**C**) Histological scoring of inflammatory infiltrates in ear wedge biopsies and livers of recipients of donor and F1 splenocytes. (**D**) Percent changes in body weight of recipients of allogeneic and F1 splenocytes. (**E**) Proliferation index of splenocytes upon mitogenic activation with concanavalin A (ConA) *in vitro* and rechallenge with naïve (DC^Naive^) and tumour-pulsed DC (DC^Neuro2a^): allografted mice (allogeneic bone marrow transplantation (allo-BMT)), recipients of F1 splenocytes alone (F1 spln.), recipients of tumour-pulsed DC (DC^Neuro2a^) alone and in conjunction with F1 splenocytes (*n*=6 in each group).

Enhanced GVT reactivity under transplant-associated lymphopenia questions whether immune reconstitution by DLI further augments anti-tumour immunity. We have recently observed that early administration of donor DC reduces tumour growth (S Ash, I Yaniv, N Askenasy, J Stein, unpublished data), whereas infusion of F1 splenocytes alone neither affect Neuro-2a growth *in vivo* nor induce GVHD as they are anergic to the host (Ash *et al*, 2009). The fast growth rates of Neuro-2a cells and the relatively fast tempo of immune reconstitution in mice impose early post-transplant immunomodulation in this experimental model. Mice bearing subcutaneous tumours were grafted with allogeneic (H2K^b^ → H2K^a^) T-cell-depleted BMCs and on day +7 were inoculated with 10^6^ donor DCs (subcutaneous) and 2 × 10^7^ donor (H2K^b^) and F1 (H2K^a/b^) splenocytes (intravenous, Figure 3A). Whereas co-administration of donor lymphocytes resulted in higher tumour sizes as compared with DC alone (*P*<0.05, Figure 3B), F1 lymphocytes suppressed tumour growth (*P*<0.01 *vs* DC alone), resulting in remarkable superior efficacy of F1-DLI (*P*<0.005 *vs* allogeneic DLI). Furthermore, early allogeneic DLI induced high-grade GVHD (*P*<0.001, Figure 3C) accompanied by weight loss (*P*<0.001, Figure 3D), whereas F1 splenocytes were devoid of GVHD activity. These data dissociate the mechanisms of GVT and GVHD, emphasising that GVHD rather impairs the efficacy of GVT reactions.

Donor DC present antigens in the context of compatible MHC to both allogeneic (H2K^b^) and haploidentical splenocytes (H2K^a/b^). The stimulatory effect of DC was evident from marked proliferative responses of lymphocytes to mitogenic stimulation at the experimental end point (4 weeks after BMT). Inoculation of tumour-pulsed DC alone enhanced the cycling rates of donor lymphocytes derived from the bone marrow of the chimeras, with a less accentuated effect after simultaneous administration of DC and F1 lymphocytes (*P*<0.05, Figure 3E). Importantly, *in vitro* rechallenge with naïve and tumour-pulsed DC generated strong proliferation (*P*<0.005 *vs* lymphocytes alone), emphasising effective DC-mediated immune activation *in vivo* as a basis for tumour growth suppression.

### Consequences of DLI and DC administration

**Figure 4:**
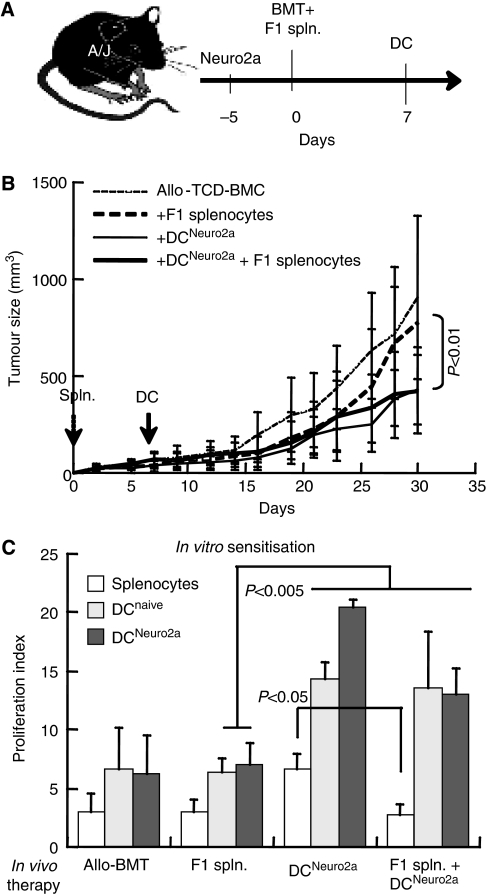
Impact of adoptive transfer of F1 lymphocytes in conjunction with allogeneic bone marrow transplantation (allo-BMT). (**A**) Experimental setting of 2 × 10^7^ F1 splenocyte (spln.) infusion concomitant with transplantation of 5 × 10^6^ T-cell-depleted bone marrow cells (TCD-BMCs) (day 0) and subcutaneous administration of 10^6^ tumour-pulsed dendritic cells (DCs) (DC^Neuro2a^) on day +7. (**B**) Tumour growth in recipients of F1 splenocytes (*n*=20), DC^Neuro2a^ (*n*=17) and their joint administration (*n*=18). (**C**) Proliferation index of splenocytes upon mitogenic activation with concanavalin A (ConA) *in vitro* and rechallenge with naïve (DC^Naive^) and DC^Neuro2a^ (*n*=5–6 in each group).

Given that the combination of F1 lymphocytes and DC suppressed tumour growth, we reasoned that fostering immune reconstitution by adoptive transfer of lymphocytes at the time of transplantation might provide additional benefit (Figure 4A). Sequential administration of F1 lymphocytes (day 0) and donor DC (day +7) conferred no significant inhibition of tumour growth (Figure 4B). Consistent with simultaneous administration of lymphocytes and DC (Figure 3E), proliferative responses were decreased by F1 lymphocyte infusion at the time of transplantation (*P*<0.05 *vs* DC alone, Figure 4C), and responsiveness was markedly increased by *in vitro* rechallenge with DC (*P*<0.005). Thus, DC administration has a stimulatory effect over lymphoid reconstitution; however, adoptive transfer of F1 lymphocytes in conjunction with BMCs does not suppress tumour growth.

**Figure 5:**
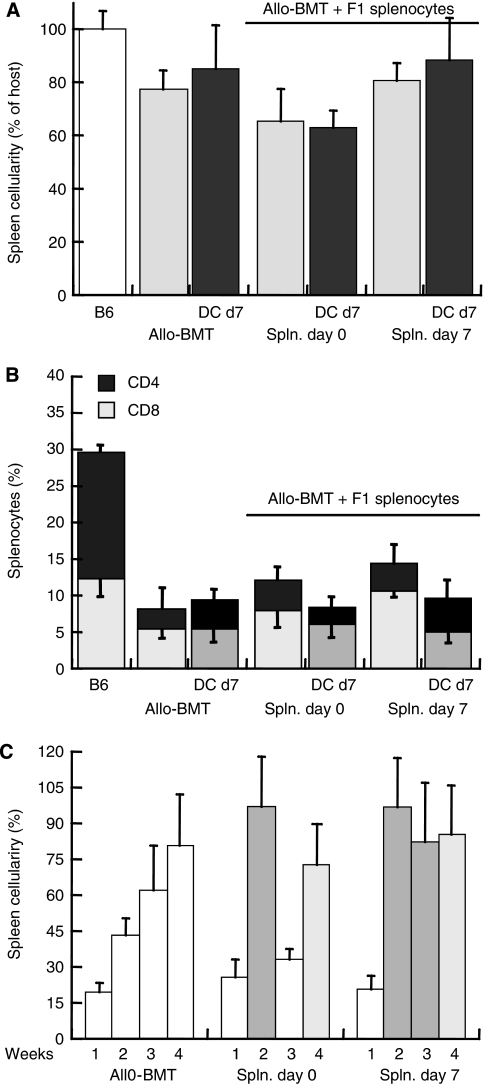
Kinetics of spleen reconstitution after bone marrow transplantation (BMT) and immunomodulation. (**A**) Percent recovery of spleen cellularity at 4 weeks after allogeneic BMT (allo-BMT) (H2K^b^ → H2K^a^), infusion of F1 splenocytes (spln.) and tumour-pulsed dendritic cells (DCs) normalised against naïve C57/BL (B6) donors (*n*=5–6 for each point). (**B**) Fractional distribution of CD4^+^ and CD8^+^ T cells in the spleens of allografted mice in the various experimental protocols. (**C**) Dynamics of splenic cellularity in allografted mice and with adoptive transfer of F1 splenocytes on days 0 and +7 as a function of time elapsing from transplantation.

Reduced proliferation of splenocytes in recipients of F1 lymphocytes might be attributed either to the timing of adoptive transfer or variable interactions with the DC. These possibilities were analysed by monitoring the immune profiles of the reconstituted mice. Quantitatively, spleen cellularity at the experimental end point of 4 weeks was reduced by F1 lymphocyte infusion (day 0) and was apparently unaffected by F1 lymphocyte infusion on day +7 (Figure 5A). Qualitatively, minor differences in splenic T-cell subsets were observed under the different treatment protocols (Figure 5B), with a trend of reduced T-cell fraction in mice infused with DC^Neuro2a^. As F1 lymphocytes are not detected in the lymphoid organs of recipients at 4 weeks after transplantation (Ash *et al*, 2009), transient engraftment might modulate lymphoid reconstitution by cells derived from the bone marrow. Administration of F1 splenocytes on day 0 resulted in transient rise in spleen cellularity that subsided during the third week, whereas infusion of F1 splenocytes on day +7 caused a more sustained rise in spleen cellularity (Figure 5C). These differences correlate with the slower growth rates of tumours attained by F1 lymphocyte infusion on day +7 as compared with day 0, showing that the kinetics of lymphoid reconstitution are important to maximise the maturing effect of DC.

### Efficacy of post-transplant immunomodulation

**Figure 6:**
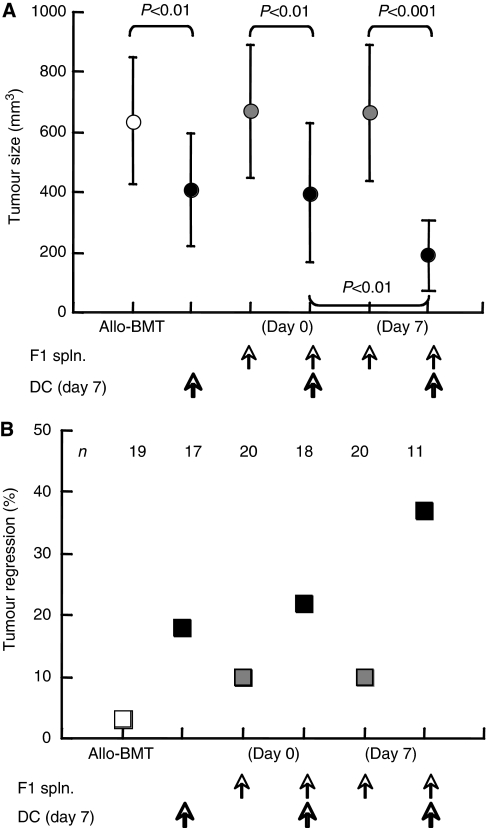
Dendritic cells (DCs) enhance GVT reactivity. (**A**) Tumour size on day 28 after transplantation of allogeneic T-cell-depleted bone marrow cells (TCD-BMCs), subcutaneous inoculation of tumour-pulsed DC on day +7 and intravenous adoptive transfer of F1 splenocytes (spln.). (**B**) Complete regression of established tumours (>60 mm^3^) expressed as percentage of the experimental groups (numbers are given for each experimental group).

The relative efficacy of the various protocols was determined by tumour size at the experimental end point and eradication of established tumours (>60 mm^3^). Although tumour-pulsed DC suppressed the growth of Neuro-2a cells under all conditions (*P*<0.01, Figure 6A), adoptive transfer of F1 splenocytes alone had no significant impact. The only additive effect was obtained by joint administration of F1 splenocytes and DC^Neuro2a^ on day +7 (*P*<0.01 *vs* DC alone, *P*<0.001 *vs* F1 splenocytes alone). Consistently, the profile of complete regression of tumours exceeding 60 mm^3^ mirrored the variations in tumour size (Figure 6B), reinforcing the validity of growth measurements. Notably, the experimental conditions were designed to maximise differences between the various experimental conditions by generating relatively large tumours. Therefore, the important component towards induction of effective GVT constitutes of donor DC infusion, which elicits potent reactions able to cause complete regression of established tumours.

## Discussion

Neuroblastoma is a potential target of GVT reactivity attained by allogeneic BMT and subsequent immunomodulation. Murine NB expresses MHC class II antigens (Ash *et al*, 2009) and elicits immune responses as efficient as A20 lymphoma in MHC-disparate mice (Asavaroengchai *et al*, 2002). However, effective GVT reactivity is mediated by cytotoxic cells derived from the bone marrow that are anergic to host MHC, indicating that tumour associated antigens are the primary targets of this reaction. Recruitment of lymphocytes reactive against the tumour is important in view of the low level expression of MHC antigens in human NB (Wolfl *et al*, 2005).

Transplant procedures associated with lymphopenia are generally associated with increased susceptibility to development of GVT reactions (Borrello *et al*, 2000; Hu *et al*, 2002). Our current data are consistent with the permissive effect of syngeneic and allogeneic BMT to evolution of anti-tumour immunity (Wrzesinski and Restifo, 2005), and the anti-tumoural activity of syngeneic transplants preceding implantation of tumours (Ash *et al*, 2009). In this study, we further demonstrate that the strain congenic to the tumour is not immunodeficient, as reconstitution of NOD/SCID mice impairs tumour growth as efficient as C57BL/6 donors. Effective tumour suppression upon transition to adaptive immunity by BMT might be particularly significant to the treatment of NB. This tumour develops in the prenatal phase from ectodermal origin (Coze *et al*, 1995; Prigione *et al*, 2004; Wolfl *et al*, 2005), and is therefore actively shielded by suppressive elements of innate immunity. This suppression is removed not only by pre-transplant conditioning, which is common to syngeneic and allogeneic transplants, but by the emerging GVT reactivity that develops during immunohaematopoietic reconstitution. Radiochemotherapy also inflicts direct injury to the tumour, facilitating selective sensitisation of the recovering immune system against tumour antigens (Asavaroengchai *et al*, 2002; Cho *et al*, 2000).

Professional processing and presentation of tumour antigens by DC elicits anti-tumour reactivity and causes complete regression of established NB tumours. Antigen-specific responsiveness is demonstrated by vigorous proliferative responses of lymphocytes upon rechallenge with tumour-pulsed DC *in vitro*. These data corroborate previous reports of potent vaccination following syngeneic BMT (Borrello *et al*, 2000; Asavaroengchai *et al*, 2002) achieved by tumour cells engineered to express co-stimulatory molecules (Jing *et al*, 2007) and cytokines (Bausero *et al*, 1995), and overexpression of cytokines in DCs (Tatsuta *et al*, 2009). A remarkable feature of DC-mediated tumour suppression was their efficacy at very low levels of T cells, reaching one-third of the steady state conditions at the experimental end point. Consistent with participation of DC in maturation of the developing immune system, the quantitative deficit was compensated by pronounced responses to antigens characteristic of lymphopenic mice as compared with T-cell-sufficient mice (Cho *et al*, 2000; Asavaroengchai *et al*, 2002; Shklovskaya and Fazekas, 2006).

In contrast to our initial assumption that early reinforcement of lymphoid reconstitution at the time of transplantation will favour GVT reactivity, simultaneous administration of F1 lymphocytes and donor DC converged to suppress tumour growth. This differential behaviour reflects distinct mechanisms of action, given that F1 lymphocytes engraft transiently and are undetected at 4 weeks after transplantation (Ash *et al*, 2009). Infusion of F1 lymphocytes at the time of BMT impaired spleen repopulation, likely through competitive homoeostatic expansion with BM-derived lymphocytes in peripheral lymphoid organs (Teshima *et al*, 2001; Zöller, 2003). In variance, immune activation by donor DC was fostered by F1 lymphocytes, cells endowed with cytotoxic activity against Neuro-2a cells (S Ash, I Yaniv, N Askenasy, J Stein, unpublished data). Therefore, transient lymphoid support conferred by DLI is beneficial to the generation of anti-tumour immunity, but the optimal timing of administration is significant (Chakraverty *et al*, 2006). Lymphocyte infusion appears to be necessary in the context of syngeneic BMT (Jing *et al*, 2009), whereas, in allogeneic transplants, DLI can have both positive and detrimental consequences. Although we applied immunomodulation early after transplantation, the toxicity of DLI is generally reduced when administered at later times (Eto *et al*, 2008; Prigozhina *et al*, 2008). Unlike the contention of reduced DC capacity to stimulate mature lymphocytes as compared with cells developing from the bone marrow (Feuerer *et al*, 2001), our data demonstrate effective transient stimulation of mature F1 lymphocytes despite dominance of the bone marrow in immune reconstitution (Ash *et al*, 2009). A transient beneficial effect of DLI has been recently documented in a case report in which infusion of *ex vivo* expanded donor CD4^+^ T cells mediated reactivity against disseminated recurrent NB (Yoshida *et al*, 2009).

Effective GVT reactivity attained by transplantation of allogeneic T-cell-depleted BMC is dissociated from the detrimental GVH disease (Anderson *et al*, 2000; Teshima *et al*, 2001; Lang *et al*, 2006; Ash *et al*, 2009). In our hands donor DC did not inflict GVHD, although their potential to enhance anti-host reactivity of immature bone marrow-derived lymphocytes and mature haploidentical (F1) lymphocytes has been demonstrated (Li and Waller, 2004; Matte *et al*, 2004; Banchereau and Palucka, 2005). Both GVT and GVH reactions are also effectively triggered by host DC (Shlomchik *et al*, 1999) and in conjunction with DLI (Chakraverty *et al*, 2006; Xia *et al*, 2006). Our data demonstrate that the tumour-suppressive reaction induced by donor DC was impaired by ongoing GVHD, suggesting little mechanistic association between these reactions. The GVHD is mediated primarily by mature donor lymphocytes that target minor tissue antigens (Ringdén *et al*, 2009), with severe toxicity in haploidentical transplants because the effectors are not rejected. It is therefore possible to dissociate between GVHD and therapeutic GVT, which is effectively attained through tumour-specific attack by host MHC-tolerant lymphocytes. Haploidentical TCD-BMT might be of benefit as a maximal possible mismatch for stimulation of the developing immune cells against malignant tissue, a clinically feasible approach when mature GVH effectors are eliminated form the donor inoculum, which can be achieved also in delayed DLI (Eto *et al*, 2008; Prigozhina *et al*, 2008; Bohana-Kashtan *et al*, 2009). The efficacy of host and donor DC in promoting the GVT reaction without imposing GVHD (Li and Waller, 2004; Chakraverty *et al*, 2006; Xia *et al*, 2006; Ghosh *et al*, 2009) suggest that non-myeloablative transplants might be equally and even more effective in generating anti-tumour immunity (Slavin *et al*, 2000).

In summary, NB is a solid tumour effectively attacked by immune reactions, which can be reinforced by post-transplant immunomodulation. As BMT becomes a standard procedure in high-risk patients, allogeneic transplants have the significant advantage of generating potent GVT reactions. Anti-tumour reactions are promptly initiated by DC at relatively early stages after transplantation under conditions of partial reconstitution of the T-cell compartment without causing GVHD, and can be augmented by DLI. These synergistic measures are significant in the competition against an aggressive and fast growing tumour such as NB, underlining the importance of optimisation of the details of the transplant procedure.
